# U.S. postdoctoral careers in life sciences, physical sciences and engineering: Government, industry, and academia

**DOI:** 10.1371/journal.pone.0263185

**Published:** 2022-02-02

**Authors:** Maya Denton, Maura Borrego, David B. Knight

**Affiliations:** 1 Center for Engineering Education, The University of Texas at Austin, Austin, TX, United States of America; 2 Department of Engineering Education, Virginia Tech, Blacksburg, VA, United States of America; Iowa State University, UNITED STATES

## Abstract

Discussions about science and engineering postdoctoral researchers focus almost exclusively on academic postdocs and their chances of eventually securing tenure-track faculty positions. Further, biological sciences dominate policy research and published advice for new PhDs regarding postdoctoral employment. Our analysis uses the Survey of Earned Doctorates and Survey of Doctorate Recipients to understand employment implications for physical sciences and engineering (PSE) and life sciences (LS) graduates who took postdoctoral positions in government, industry, and academic sectors. We examine postdoc duration, reasons for staying in a postdoc, movement between sectors, and salary implications. There is considerable movement between employment sectors within the first six years post-PhD. Additionally, postdocs in PSE are shorter, better paid, and more often in nonacademic sectors than postdocs in LS. These results can help science and engineering faculty discuss a broader range of career pathways with doctoral students and help new PhDs make better informed early career decisions.

## Introduction

Many doctoral recipients in the life sciences, physical sciences, and engineering enter postdoctoral employment following their graduation, and the number of postdoctoral positions continues to rise. The percentage of postdoctoral positions within initial post-PhD employment varies by field, with the highest rate in the biological sciences at 80% [1]. Among 2012 Survey of Earned Doctorates respondents with job offers at graduation, two-thirds of biological sciences PhDs entered postdoctoral employment, followed by one-half of physical scientists and one-third of engineers [2]. The most rapid growth in number of postdoc positions is occurring in fields with fewer postdocs, i.e., engineering and social sciences [2]. Between 2000 and 2008, the number of engineering postdocs increased by 64% and social sciences postdocs by 32%, compared to 22% in the life sciences and 9.8% in the physical sciences [3]. Recent reports show that the postdoc workforce in 2018 was slightly above 37,500 in science fields and almost 8,000 in engineering, with 46.4% of science postdocs and 33.6% of engineering postdocs having U.S. citizenship [4].

Given the prevalence of postdoc positions as part of the post-PhD pathway and their continued growth, it is becoming increasingly important to understand how being employed as a postdoc relates to an individual’s career trajectory and future employment. Many prior studies assume that all postdoctoral researchers aspire to a tenure-track faculty career at a research-intensive institution [1, 5, 6] or focus exclusively on the experiences of academic postdocs [2]. However, prior research has found that only 10% of PhDs [7] and 17% [8] to 21% of postdocs [1] eventually enter into a tenure-track faculty position. The growth in postdocs over the past few decades has far outpaced growth in tenure-track faculty positions [1, 2], raising the question of whether time spent as a postdoc is worthwhile in terms of preparation for a future tenure-track role, or how the postdoc can be worthwhile training for other career options. By 2011, the number of new faculty positions in science and engineering was seven times less than the number of new PhD graduates [9]. This changing landscape in academia is partially due to the (over)reliance on postdocs and graduate students as research labor in higher education [10], which may be driven by the increasingly team-based and collaborative structure of research [11]. Despite the frequency of postdocs entering non-academic pathways, we know very little about the career paths of postdocs who end up in permanent positions in government, industry and non-tenure track positions in academia.

There are also growing concerns that groups already marginalized in science and engineering fields are disproportionately impacted by the increasing prevalence of postdoctoral researcher positions [2, 12–14]. Studies have found that postdocs are generally not very satisfied in their jobs and that their job satisfaction depends heavily on the quality of mentoring they receive [15–17]. Although postdoc positions are often encouraged for graduates to gain further research experience and increase their publications, the research productivity increase seen among postdocs, in terms of peer-reviewed publications, lasts for only 3 years [18]. There are also lasting salary implications in taking a postdoc, as postdocs earn much less than their peers who take permanent positions [2, 19–21], and this pay gap can persist for as long as 15 years [1]. However, it can be difficult to account for geographical differences in cost-of-living in such salary analyses, which may influence salary outcomes [22]. Historically (and problematically), women and doctorates of color have been less likely to attain tenure-track positions [12, 14, 23, 24]. Studies have found that research productivity and institutional prestige (but only for the highest-ranked institutions) partially explain who lands sought-after tenure-track faculty positions [18, 23]. Postdocs may experience disillusionment upon realizing that the reputation of their training institutions and supervisors alone do not guarantee them a faculty position [6], indicating that most postdocs do not think they will be in the majority who ultimately do not obtain a tenure-track position.

However, focusing solely on academic postdocs as a precursor to tenure-track positions ignores the career pathways of most science and engineering PhD recipients. Many factors influence the career decisions of recent doctorates, including their personal values or goals [25], familial obligations [24], and workplace structure [25]. In particular, women in science begin to consider how familial obligations will influence their career plans early on in graduate school [26]. Additionally, doctorates often take postdoc positions “without a clearly defined career goal” [25] and open to careers in different sectors. A decade after receiving their degrees, 46% of science and engineering PhDs were employed in industry and 11% held a government position [24]. Dietz and Bozeman [27] found that among academic scientists and engineers supported by university research centers, half had worked for industry and one-quarter had worked in government. For their first positions post-PhD, nearly half had begun their careers outside of academia; 33% started in industry and 15% in government. They found that spending one’s entire career in academia positively impacted publication productivity, but that spending time in industry positively impacted patent productivity [27]. Therefore, not only is movement between sectors relatively common, but it may hold some advantages for both employees and employers. Given the increasing pressure on faculty to cultivate new sources of research funding, experience in industry or government settings may help such postdocs develop important skills and contacts for future academic positions. However, there can be drawbacks for academic postdocs who switch sectors. Academic postdocs who seek industry positions are less competitive than other candidates and often need to be retrained [28], and too many years in an academic postdoc can be viewed by recruiters as a lack of interest in industry [29].

The National Academies report that 11% of postdocs are employed outside of academia [2]; however, there is limited information about the experiences of such postdocs and their career paths are less clear than those of academic postdocs. Reports and advice columns suggest that postdocs at national labs and in industry (as compared to universities) are better paid, remain in postdoc positions for less time (2–3 years as opposed to 5 or more) and have clearer paths to full-time employment with their postdoc employers [2, 29]. Thus, postdoctoral positions in U.S. government (e.g., national labs) and industry may have some advantages in making researchers more competitive for permanent employment in that sector [28]. In both settings, the teamwork and management style, access to cutting-edge technologies and equipment, and potential for permanent employment are considered advantages [28–30]. National laboratories are often modeled similarly to academic research, with a focus on long-term research projects and less profit-driven, while industry has a greater emphasis on production and consumers. Therefore, the government sector may be better equipped and more willing to create postdoc roles. There are also potential drawbacks associated with employment in a non-academic postdoc. In government and industry settings, opportunities to teach and mentor students are limited, and postdocs may have less ownership and flexibility in their projects [30]. In industry, proprietary restrictions may limit postdocs’ ability to publish their research [30], although some companies expect their postdocs to publish [28], and postdocs are less likely to get experience writing funding proposals [28].

Previous National Academies reports call for better advising of graduate students and postdoctoral researchers about career options in their fields, particularly around alternatives to tenure-track faculty positions at research-intensive universities. Our paper addresses the concern that "current data on the postdoctoral population, in terms of demographics, career aspirations, and career outcomes are neither adequate nor timely" [2] by providing data on recent career outcomes of postdocs in the life sciences, physical sciences, and engineering. Polka, Krukenberg and McDowell [31] call for additional reports on the STEM workforce, particularly for graduate students and postdocs, because it is unclear whether there actually is a labor shortage in STEM that needs to be filled. In this paper, we aim to examine and share a more complete picture of postdoctoral career trajectories, including government, industry and academic employment sectors; movement between sectors; and salary implications. Our research question follows:

What are the early career outcomes for postdocs in the life sciences, physical sciences and engineering across employment sectors (i.e., academia, government, and industry) in terms of sector movement and salary implications?

Using the Survey of Earned Doctorates (SED) [32], we examined a recent decade of doctoral recipients (2009–2018) in the physical sciences and engineering (PSE) and life sciences (LS). The SED includes data for nearly every doctoral recipient from U.S. institutions (n = 2,269,267). To follow respondents’ resulting career paths, we matched SED data with the Survey of Doctorate Recipients (SDR) datasets [33]; the SDR samples a subset of PhD recipients every two years (n = 85,739 for SDR 2017). In our analysis, we considered employment sectors of postdocs, postdoc length, reasons for choosing a postdoc, movement between employment sectors, and salary differences between sectors. Both U.S. and non-U.S. citizens are included in the analyses. After running analyses separately for the life sciences, physical sciences, and engineering, we combined physical sciences and engineering into one category because of similarities between the two fields. We did not include mathematics because of the lower numbers of postdocs overall and in U.S. non-academic positions. For postdoc analyses, we include the five most common employment sectors: academic, government, industry, non-U.S., and non-profit. For analysis of permanent employment, we focus on the three largest employment sectors: academic, government, and industry.

## Materials and methods

### Data source

We utilized data from two existing national datasets: the Survey of Earned Doctorates (SED) and the Survey of Doctorate Recipients (SDR). We connected respondents’ data in the SED and SDR through linked participant ID numbers since our analysis focuses on specific timepoints directly following graduation (SED) and the first eight years of employment (SDR). From the SED, we used questions related to employment type, employment sector, salary, and STEM field. From the SDR, we used questions related to employment type, employment sector, and salary at subsequent years.

The SED is a census that has been administered annually since 1957 to all graduating students who received a research-based doctoral degree from a U.S. institution. In 2018, 92.1% of all students who graduated with a research-based doctorate between June 2017 and 2018 completed the survey. The National Center for Science and Engineering Statistics (NCSES) and five additional federal government agencies sponsor SED administration [32]. Survey questions relate to respondents’ doctoral degrees, experiences during graduate school, plans for post-graduation employment, and personal background or demographic information. The SDR is a national survey administered every two to three years since 1973 to a sample of all prior recipients of a research-based doctoral degree in science, engineering, or health from a U.S. institution. Therefore, the SDR and matched SED-SDR data include substantially fewer participants than the SED. NCSES and the National Institutes of Health (NIH) sponsor SDR administration [33]. Survey questions relate to current employment, including job role and work activities, past employment, additional education obtained, and demographic information. We mention specific codes from the SED and SDR in the following sections, rather than question numbers, as the codes remains constant while questions can change from year-to-year.

### Participants

All participants in our dataset completed the SED between July 1, 2008 and June 30, 2018 (FY2009–2018). We chose the most recent decade of data to which we had access at the time of analysis, ensuring a large sample size. To analyze postdoctoral employment at graduation, we included participants who completed the SED at time of graduation; received a doctorate in the physical sciences, life sciences, or engineering (including computer science); and obtained a postdoctoral position at graduation. For analysis involving postdoc length, reasons for remaining in a postdoc, movement between sectors, and salary, we linked SED and SDR data for respondents who completed the SED between 2009 and 2018 and the SDR in 2013, 2015, and/or 2017. Both U.S. and non-U.S. citizens are included in the analyses. For analysis of permanent employment at graduation, we similarly included participants who completed the SED at the time of graduation; received a doctorate in the physical science, life sciences, or engineering; and obtained a permanent position at graduation. Detailed descriptions of participant subsets for each analysis are available below in Table 1 and upon request.

**Table 1:** Participant samples used for each analysis.

Analysis	Dataset	Description	Employment sectors
Postdoctoral employment sectors (n = 81,539)	SED	Received a doctoral degree in LS or PSE between 2009 and 2018 and took a postdoc position at graduation (i.e., indicated took postdoc in their SED responses)	Academic, government, industry, non-U.S., non-profit
Permanent employment (n = 71,880)	SED	Received a doctoral degree in LS or PSE between 2009 and 2018 and took a permanent position at the time of graduation (i.e., indicated did not take a postdoc in their SED responses)	Academic, government, industry, non-U.S., non-profit
Postdoc length (n = 11,008)	SED, SDR	Respondents in LS and PSE who were in Years 2 through 8 of their employment following employment in a postdoc at graduation	Academic, government, industry, non-U.S., non-profit (at graduation)
Reasons for remaining in a postdoc (n = 1,313)	SED, SDR	Respondents in LS and PSE who took a postdoc at graduation and held a postdoc position two years later	Academic, government, industry, non-U.S., non-profit (at Year 2)
Movement between sectors (n = 1,985)	SED, SDR	Respondents in LS and PSE who took a postdoc at graduation and held permanent employment at Year 5–6	Academic, industry, government (at graduation and at Year 5–6)
Initial Salaries (n = 58,205)	SED	Respondents in LS and PSE who took a postdoc at graduation and filled out their starting salary information	Academic, government, industry, non-U.S., non-profit (at graduation)
Salary gaps in subsequent permanent employment (n = 2,631)	SED, SDR	Respondents in LS and PSE who took a postdoc or permanent employment at graduation, held permanent employment at Year 5–6, and filled out their Year 5–6 salary information in the SDR	Academic, government (at graduation); Academic, industry, government (at Year 5–6)

In the *postdoc length* analysis, respondents were removed if they were in Year 0 or 1 at the time of SDR response. Due to the nature of the longitudinal sampling, it is possible that some individual respondents are represented multiple times and others are represented only once. The question used in the *reasons for remaining in a postdoc* analysis is included on the SDR but not the SED. All respondents who initially took a postdoc and transitioned to permanent employment within two years were removed from this dataset. Our analysis on *reasons for remaining in a postdoc* is based on the type of postdoc the respondents held at Year 2, rather than the initial type of postdoc respondents accepted at graduation. In the *movement between sectors* and *salary gaps* analysis, Year 5–6 refers to the fifth- or sixth-year following graduation, with two years included to account for biennial administration of the SDR survey. Per our data agreement, we cannot report results for fewer than 5 individuals and removed those individuals from our dataset, specifically in the *salary gaps* analysis.

### Data treatment

All variables were derived from responses to items on the SED or SDR. The following sections describe how we derived variables based on specific codes from the surveys, including employment type, PhD field, and employment sector. Whenever possible, we include details involving the survey codes, response options, and aggregation of response options. We used fiscal year, rather than calendar year, for determining all time periods, including FY for PhD graduation and years since PhD.

#### Employment type at graduation

For all analyses that involved respondents with postdoctoral employment at graduation, we separated doctoral recipient employment into the two categories of *postdoc* or *permanent employment*. Based on SED questions, we defined postdoctoral employment as any temporary employment, with a pre-specified endpoint. Similarly, we defined permanent employment as any non-postdoctoral role, which does not have a pre-specified endpoint. To determine employment status of the respondents, we used SED code PDOCSTAT. We removed respondents from that dataset who had not yet committed to post-graduate employment, including those who were still negotiating with organizations, seeking positions with no prospects, continuing in other full-time degree programs, or had no plans to work or study. Respondents remained in the dataset if they had made a commitment to employment following their graduation, including returning to or continuing in predoctoral employment. We used SED code PDOCPLAN to identify the employment type, i.e., postdoc or permanent employment, for the respondents. If respondents selected postdoc fellowship or postdoc research associateship, their employment status was designated as *postdoc*. We excluded respondents who were continuing in additional training, such as traineeships and internships/clinical residency, due to the difference between postdoc positions and such programs. All other respondents were assigned a label of *permanent employment* for their employment type, including military service and other and unspecified employment.

#### Employment type in years following graduation

For analysis involving employment type in the years following graduation, we removed respondents who were no longer in the workforce, using SDR code WRKG to determine their workforce status. We designated the two employment types as *postdoc* and *permanent employment*. SDR code PDIX is associated with whether the respondents’ current job is a postdoc position, which we used to determine their current employment type during the first eight years of employment. If the respondent answered yes to SDR code PDIX, their employment type was designated as *postdoc*, while an answer of no designated a label of *permanent employment*.

*PhD discipline*. Respondents indicated their PhD discipline in SED code PHDFIELD. We removed all non-STEM individuals from the overall dataset, as well as health and social sciences. The remaining STEM disciplines were categorized as life sciences, physical sciences, engineering, and mathematics. Following initial analysis, mathematics was removed from our dataset because of its smaller sample size and different behavior related to non-academic postdocs. Physical sciences and engineering (including computer science) yielded similar results from our analyses and were subsequently combined into one category. All analyses were conducted for both the *life sciences (LS)* and *physical sciences and engineering (PSE)*.

*Race*, *sex*, *and citizenship status*. Respondents answered two questions about their race/ethnicity in the SED, resulting in the following SED codes: HISPANIC (Hispanic or Latino), AMERIND (American Indian or Alaska Native), HAWAIIAN (Native Hawaiian or other Pacific Islander), ASIAN (Asian), BLACK (Black or African American), and WHITE (White). We categorized participants as part of an underrepresented racial group (URG) if they identified as Hispanic or Latino, American Indian or Alaska Native, Native Hawaiian or other Pacific Islander, and/or Black or African American. We categorized participants as Asian if they identified as Asian or as Asian and White. We categorized participants as White if they identified as White and not as any other racial or ethnic groups. Respondents indicated their sex in SED code SEX, with the options of identifying as male or female. Respondents recorded their citizenship status in the SED, collected as SED code CITIZ. We categorized participants as U.S. citizens if they indicated they were a naturalized or native-born U.S. citizen or a non-U.S. citizen with a permanent U.S. resident visa. Participants were categorized as non-U.S. citizens if they indicated they held a temporary U.S. visa.

#### Employment sector at graduation

Employment sector at graduation (SED code PDEMPLOY) contained fourteen response options, which we condensed into the categories of *academic*, *government*, *industry*, *non-U*.*S*., and *non-profit*. Respondents who held initial positions in K-12 educational institutions, two-year institutions, self-employment, or other were filtered out of the dataset. Academia includes employment at U.S. four-year colleges or universities other than medical school, U.S. medical schools, and U.S. university-affiliated research institutes. Government includes employment in the U.S. federal, state, or local levels of government. Industry includes employment in industry or business. Non-U.S. includes employment in non-U.S. educational institutions or governments. Non-profit includes employment in not-for-profit organizations. With the given SED questions, permanent employment within academia cannot be further specified to tenure-track or non-tenure-track positions at graduation; this information is available from SDR.

#### Employment sector in years following graduation

SDR codes NEDTP, EMED, and TENSTA were used to determine employment sector at timepoints following graduation, which we condensed into the four categories of *academic (tenure-track)*, *academic (non-tenure-track)*, *government*, and *industry*. Respondents who held non-academic employment in the categories of non-U.S., non-profit, self-employment, or other, as indicated by code NEDTP, were removed from the dataset.

Code NEDTP reported respondents’ employment sectors for non-academic sectors, which we condensed into the following two categories: *government* and *industry*. We included respondents with employment in the local, state, and federal government and those in military service within the government category. Code EMED indicated whether respondents are working at an educational institution, which we used to assign *academic* as their employment sector. Respondents could have employment listed as both academic and government or industry, based on their responses to codes NEDTP and EMED. If this was the case, we assigned academic as their employment sector and removed respondents from the other category. We used code TENSTA, which asked about tenure status, to separate out respondents who were in tenure-track roles within academia, assigning *academic (tenure-track)* and *academic (non-tenure-track)* as their own categories.

#### Reasons for remaining in a postdoc

We drew from SDR question A24, which asked respondents to check all reasons for being in a postdoc at the current time from a list of six options. The six codes associated with question A24 were as follows: PDTRAIN (additional training in PhD field), PDTROUT (training in area outside of PhD field), PDPERPL (work with a specific person or in a specific place), PDEMPL (other employment not available), PDEXPECT (postdoc generally expected for a career in this field,) and PDOTHER (some other reason).

#### Salary

SED code SALARYV asked respondents for their basic annual salary for their principal job, which we used to calculate median salary at graduation. SDR code SALARY asked respondents for their basic annual salary for their principal job, which we used to calculate median salary at Year 5–6. Using the Consumer Price Index (CPI) [34], we converted all respondent salaries to 2018 dollars.

### Data analysis

We calculated descriptive statistics, including counts, percentages, medians, and weighted averages. All data analysis was completed in RStudio and Excel, and each of the described analyses occurred for both LS and PSE. We computed counts and percentages for respondents with postdocs and permanent employment at graduation by sex (male, female), race (URG, Asian, and White), and citizenship status (U.S., non-U.S.) in our analysis of *employment type*; for postdoc employment sector at graduation overall and by sex (male, female), race (URG, Asian, and White), and citizenship status (U.S., non-U.S.) in our analysis of *postdoctoral employment sectors*; on reasons respondents remained in a postdoc by employment sector in our analysis of *reasons remaining in a postdoc*; and on postdoc employment sector at graduation and permanent employment sector at Year 5–6 in our analysis of *movement between sectors*. We found median salaries for postdoctoral employment at graduation and permanent employment at Year 5–6 by employment sector for analysis of *initial salaries* and *salary gaps*.

We conducted additional statistical analysis in the form of chi-square tests when relevant, specifically for analyses of *employment type* and *postdoctoral employment sector*. Within each field, we ran chi-square tests by sex and race to determine if there were statistical differences in how frequently doctorates entered specific employment types and postdocs were in specific employment sectors by demographics. For the *movement between sectors* analysis, we used Sankey diagrams to format count data into a visual representation of employment sector movement from graduation to Year 5–6. The diagrams display the various employment sector pathways when respondents accepted a postdoc offer at graduation and held permanent employment at Year 5–6. The thickness of each pathway in a diagram corresponds with the number of respondents moving between individual sectors. Strands corresponding to values of n < 5 were removed from the diagrams per our data licensing agreement. For the *salary gaps* analysis, salary gaps and surpluses were calculated based on the overall median salary for respondents starting in academic and government positions for PSE ($94,500) and LS ($81,600). Per our data licensing agreement, we cannot report results for fewer than 5 individuals and removed those combinations from our dataset.

For the *postdoc length analysis*, we first calculated descriptive statistics (counts and percentages) on whether respondents remained in a postdoc position in subsequent years. Next, we calculated a weighted average to estimate average postdoc length for each employment sector. Weighted averages of length of postdoc were calculated using the number of SDR respondents in each year who indicated they were still in a postdoctoral position. For example, we assumed that all respondents who started in a postdoc and held permanent employment at Year 2 had a postdoc length of one year. To account for varying response rates, we standardized the number of respondents by multiplying the response rate for a given year by the number of respondents in year 2. In a few cases, the percentage of respondents in a postdoc position increased in later years, e.g., more 8^th^ year postdocs than 7^th^ year postdocs. In these cases, we considered the longest postdoc length in our calculations, assuming that no respondents left in the earlier year, so all respondents were weighted in the later year. While these methods do not yield exact values for postdoc length, we consider them sufficient to calculate an estimated average postdoc length per field and sector and to draw conclusions regarding broad trends.

### Limitations

The SED and SDR do not include questions about research productivity, such as number of journal publications. Therefore, we were unable to incorporate productivity as a variable in our analysis. We refer to biological sex as “male” or “female”, as the SED asks respondents “Are you male or female?” with the two options. This is a limitation of the dataset in that there are no questions about gender identity and multiple options are not provided (i.e., non-binary, trans man or woman, etc.). All data collected in the SED and SDR are self-reported, meaning that respondents may choose not to respond to any potentially sensitive questions.

## Results

First, we considered the total number of PSE and LS postdocs and their respective employment sector breakdowns (Table 2). Over the past decade, 45.0% of PSE (n = 40,564) and 64.8% of LS (n = 40,975) doctoral recipients took postdoctoral employment upon graduation, indicating the prevalence of a postdoc following completion of science and engineering PhDs. The majority of postdocs were employed within the academic sector: 71.8% of PSE and 79.9% of LS postdocs. Over one-quarter of PSE postdocs and one-fifth of LS postdocs were in other sectors, most frequently government and non-U.S. organizations.

**Table 2:** Percentage of postdocs and estimated length of postdoc by field and employment sector from 2009 to 2018.

	Physical sciences/engineering (n = 40,564)		Life sciences (n = 40,975)		Mathematics (n = 5,050)
	Percent in sector (%)	Length of postdoc (yr)	Percent in sector (%)	Length of postdoc (yr)	Percent in sector (%)
Academic	71.8	2.40	79.9	3.31	71.8
Government	12.0	2.16	8.0	2.67	3.9
Industry	3.3	1.28	2.1	1.63	1.0
Non-U.S.	10.5	3.06	5.2	3.58	21.8
Non-profit	2.4	2.38	4.8	3.20	1.6

We further analyzed postdoc employment sector for each field by sex and race (Table 3), finding that postdoc employment sectors differed by sex for both PSE (χ^2^(4) = 23.4, *p* < 0.001) and LS (χ^2^(4) = 33.3, *p* < 0.001). In both fields, individuals who identified as female were employed as postdocs in the government sector more frequently than individuals who identified as male. Postdoc employment sector also differed by race for both PSE (χ^2^(8) = 551.2, *p* < 0.001) and LS (χ^2^(8) = 155.3, *p* < 0.001). Asian postdocs were employed more frequently in the academic sector when compared to White and URG postdocs, particularly in PSE. In contrast, White and URG postdocs were employed more frequently in the government sector when compared to Asian postdocs in both fields.

**Table 3:** Employment sector of postdocs (%) by sex and race from 2009 to 2018.

	Physical sciences and engineering					Life sciences				
	Sex		Race			Sex		Race		
	Male	Female	White	Asian	URG	Male	Female	White	Asian	URG
	*n = 29992*	*n = 10572*	*n = 20504*	*n = 16443*	*n = 3093*	*n = 19649*	*n = 21323*	*n = 23396*	*n = 12191*	*n = 4984*
Academic	72.0	71.0	68.1	76.8	69.6	80.4	79.4	79.1	82.1	78.0
Government	11.7	12.8	14.9	8.1	13.2	7.2	8.7	8.7	5.9	9.8
Industry	3.3	3.2	2.9	3.8	3.3	2.1	2.1	1.8	2.5	2.4
Non-U.S.	10.7	10.1	11.6	8.8	11.7	5.5	5.0	5.5	4.5	5.6
Non-profit	2.3	2.9	2.5	2.4	2.1	4.8	4.8	4.8	5.0	4.3

We also found differences in employment outcomes for PSE and LS doctoral recipients who took permanent employment at the time of graduation. Fewer LS doctoral recipients took permanent employment (35.2%; n = 22,261) compared to PSE doctoral recipients (55.0%; n = 49,619). These employment outcomes significantly differed by sex for PSE (χ^2^(1) = 136.2, *p* < 0.001) and LS (χ^2^(1) = 205.3, *p* < 0.001), as well as by race for PSE (χ^2^(2) = 74.3, *p* < 0.001) and LS (χ^2^(2) = 352.7, *p* < 0.001) (Table 4). Individuals who identified as female were more frequently employed as postdocs in PSE compared to individuals who identified as male, whereas individuals who identified as female were less frequently employed as postdocs in LS. In PSE, White doctorates were more frequently employed in postdocs than URG and Asian doctorates. However, LS Asian doctorates were more frequently employed in postdocs than URG and White doctorates.

**Table 4:** Percentage of employment type by sex and race from 2009 to 2018.

	Physical sciences and engineering					Life sciences				
	Sex		Race			Sex		Race		
	Male	Female	White	Asian	URG	Male	Female	White	Asian	URG
	*n = 68339*	*n = 21842*	*n = 44200*	*n = 37898*	*n = 3093*	*n = 29*,*000*	*n = 34*,*232*	*n = 37*,*370*	*n = 17*,*250*	*n = 7958*
Postdoc	43.9	48.4	46.4	43.4	44.9	67.8	62.3	62.6	70.6	62.6
Permanent	56.1	51.6	53.6	56.6	55.1	32.2	37.7	37.4	29.4	37.4

We examined employment type at graduation by citizenship status, finding variation between the two disciplines. Within PSE, non-U.S. citizens (45.6%; n = 43,397) entered postdoc employment at similar rates to U.S. citizens (44.5%; n = 46,536). However, within LS, a greater number of non-U.S. citizens entered postdoc employment (71.4%; n = 17,529) compared to U.S. citizens (62.3%, n = 45,580). There were also differences in postdoc employment sector by citizenship status (Table 5). Sector differences included non-U.S. citizens more frequently in academic (PSE) and non-U.S. (PSE and LS) sectors, while U.S. citizens were more frequently in government (PSE and LS, but more pronounced in PSE).

**Table 5:** Employment sector of postdocs (%) by citizenship status from 2009 to 2018.

	Physical sciences and engineering		Life sciences	
	U.S.	Non-U.S.	U.S.	Non-U.S.
	*n = 20*,*689*	*n = 19*,*782*	*n = 28*,*388*	*n = 12*,*510*
Academic	67.5	76.2	79.7	80.2
Government	17.1	6.7	9.3	5.0
Industry	3.0	3.6	2.0	2.3
Non-U.S.	9.9	11.2	4.2	7.7
Non-profit	2.6	2.3	4.8	4.8

Then, we estimated the length of postdoctoral training by employment sector for PSE and LS (Table 2), based on the proportion of respondents remaining in a postdoc between 2 and 8 years after their initial postdoc employment at the time of PhD completion. The estimated average length of a PSE postdoc ranges from 1.3 years (industry) to 3.1 years (non-U.S. positions), slightly shorter than estimated average lengths of LS postdocs in each sector (1.6 to 3.6 years). As previously suggested [2, 29], government, industry and non-profit postdocs remained in a postdoc position for less time than academic postdocs. All length estimations do not consider whether respondents completed one or multiple postdoc positions.

We also examined the reasons individuals chose to remain in a postdoctoral position following initial postdoctoral employment. Using SDR responses collected 2 years after PhD completion, continuing PSE (n = 547) and LS (n = 766) postdocs across sectors cited a range of reasons for engaging in postdoctoral training (Table 6). In both fields, respondents chose to remain in postdoctoral employment for further training or viewed it as being an expectation of their field. The least frequent response was that no other employment was available, and so results suggest that postdoctoral employment generally is considered part of a normal career pathway for both PSE and LS PhDs. We note that government and industry postdocs in PSE more frequently indicated that no other employment was available; industry percentage cannot be reported because of the small sample size, per our data licensing agreement. In terms of reported employment availability by citizenship status, there were mixed results between disciplines, with comparable percentages between U.S. citizens (25.8%, PSE; 21.3%, LS) and non-U.S citizens (22.5%, PSE; 25.7% LS) indicating that no other employment was available.

**Table 6:** Reasons (%) for remaining in a postdoc at Year 2 for life sciences, physical sciences and engineering.

Reason (%)	Government		Industry		Academic		Non-U.S. positions		Non-profit	
	PSE *n = 51*	LS *n = 72*	PSE *n = 13*	LS *n = 16*	PSE *n = 394*	LS *n = 541*	PSE *n = 47*	LS *n = 47*	PSE *n = 42*	LS *n = 90*
Training	76.5	86.1	76.9	81.3	75.1	74.7	68.1	72.3	66.7	78.9
Outside training	49.0	48.6	38.5	50.0	45.9	46.8	42.6	44.7	45.2	47.8
Person/place	70.6	65.3	61.5	68.8	64.5	60.8	57.5	85.1	71.4	56.7
Not available	39.2	25.0	-	-	22.8	23.5	23.4	19.2	19.1	20.0
Expected	86.3	84.7	6.15	87.5	76.7	83.9	87.2	87.2	69.1	93.3
Other	19.6	9.72	-	-	7.61	6.84	-	-	-	5.56

**Fig 1:**
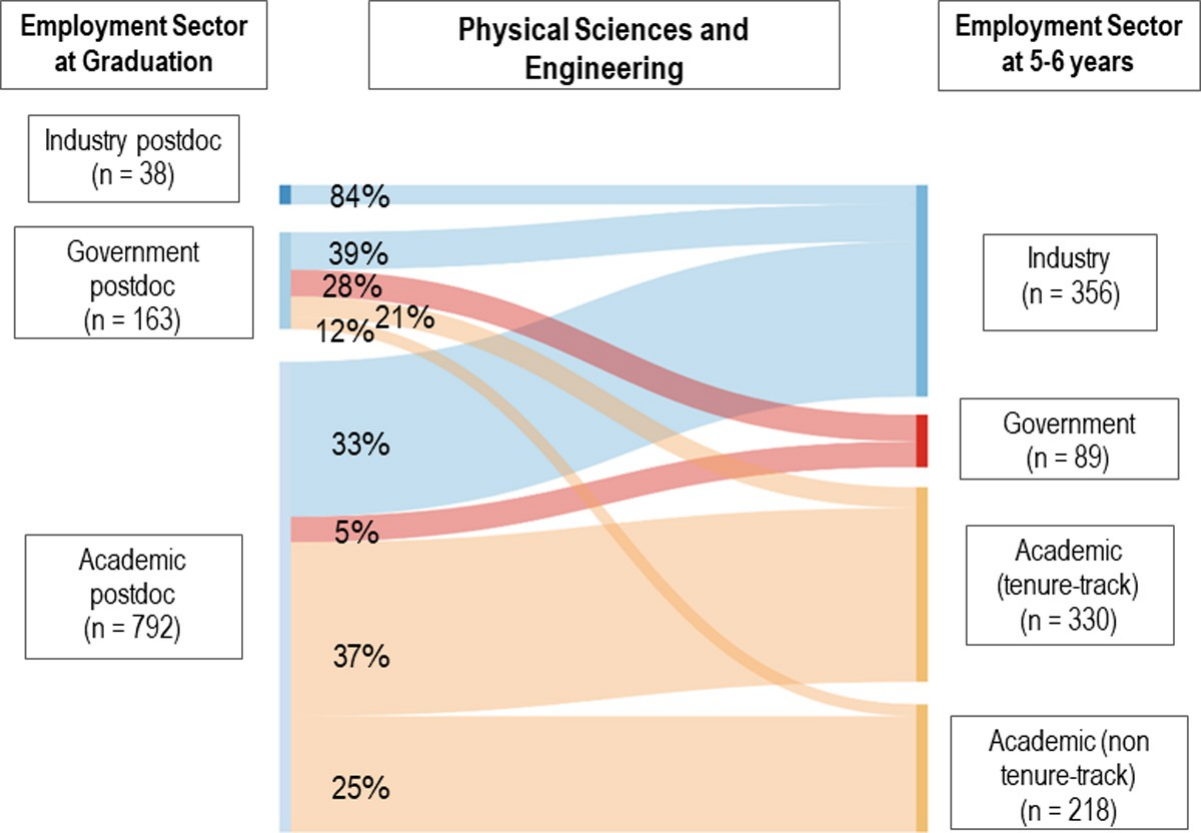
Sankey diagram of physical sciences and engineering postdoc movement to permanent employment.

**Fig 2:**
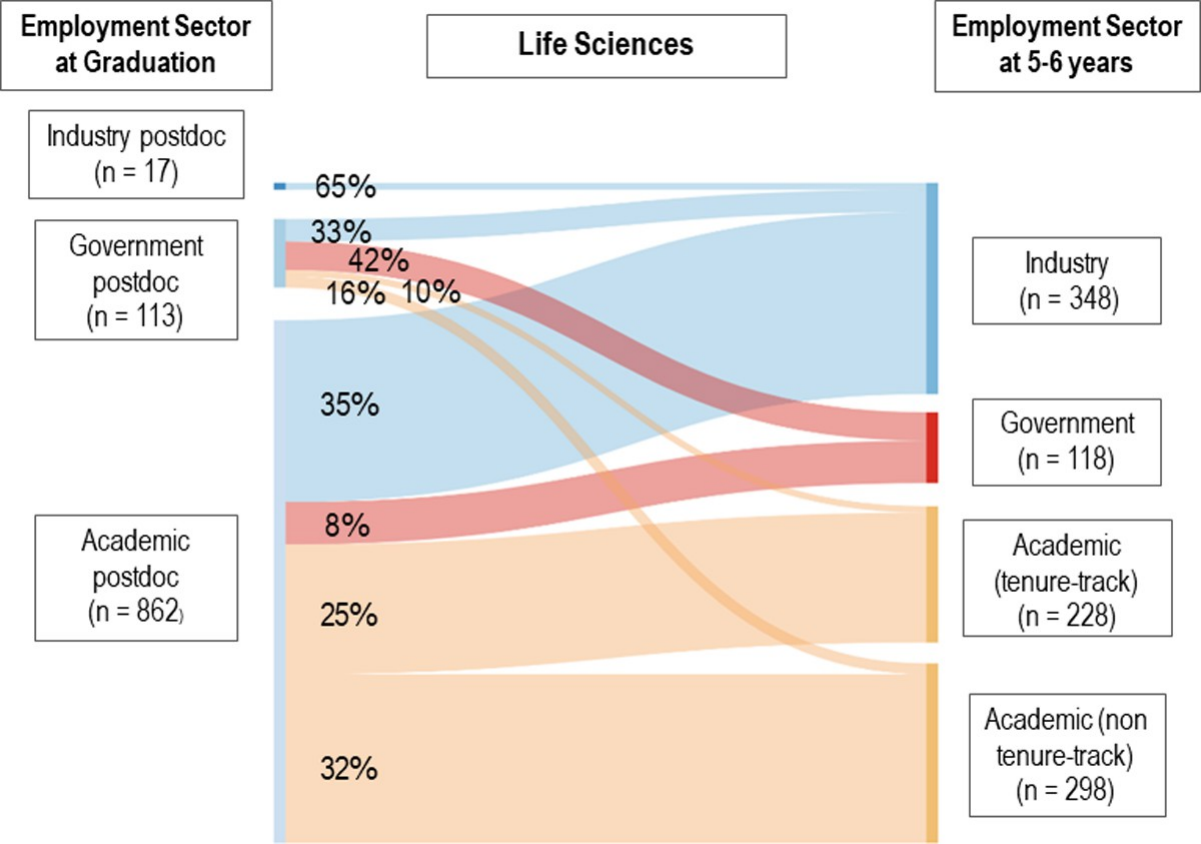
Sankey diagram of life sciences postdoc movement to permanent employment.

How frequently do postdoctoral researchers move to a different employment sector? Figs 1 and 2 illustrate the movement of academic, government and industry PSE (Fig 1) and LS (Fig 2) postdocs to permanent positions in these three sectors 5–6 years after completing their PhDs. Those remaining in a postdoc at 5–6 years were removed from the analysis (n = 94, 8.6%, PSE; n = 288, 22.5%, LS). As expected (28), postdocs most frequently found permanent employment in the same sector, particularly industry (84.2% PSE, 64.7% LS). However, government postdocs dispersed into all three permanent employment sectors, similar to the trajectory of academic postdocs. Among PSE government postdocs, 21.5% moved to tenure-track faculty positions, 12.3% to other academic permanent employment, 27.6% to government, and 38.7% to industry. Based on our analysis, only one-third of PSE postdoctoral researchers and less than one-quarter of LS postdocs ultimately transitioned to tenure-track positions within 5–6 years.

Visual representation of the movement between industry, government, and academic sectors for individuals who held postdoctoral employment at graduation and permanent employment at 5–6 years (n = 993, PSE). Strand widths correspond to the number of individuals in a pathway. Strands corresponding to values of n < 5 were removed; therefore, industry postdoc strands will not add to 100% for both PSE and LS diagrams.

Visual representation of the movement between industry, government, and academic sectors for individuals who held postdoctoral employment at graduation and permanent employment at 5–6 years (n = 992, LS). Strand widths correspond to the number of individuals in a pathway. Strands corresponding to values of n < 5 were removed; therefore, industry postdoc strands will not add to 100% for both PSE and LS diagrams.

Although government postdoc dispersal patterns are more pronounced in PSE, they also occur within LS. Comparably, among LS government postdocs, 9.7% move to tenure-track faculty positions, 15.9% to other academic permanent employment, 41.6% to government, and 32.7% to industry permanent positions. For LS academic postdocs, 24.8% move to tenure-track, 32.3% to other academic, 8.1% to government, and 34.8% to industry. Therefore, 23.0% of LS postdocs obtained tenure-track employment within 5–6 years. We ran a logistic regression model on sector movement (see S1 Table in S1 Appendix), finding that sector at graduation and race were both statistically significant in predicting sector movement between graduation and Year 5–6. However, the variance explained by the model is less than 10%.

Another important consideration of postdoctoral career pathways is salary. Our analyses show that median starting salaries for PSE postdocs in government and industry are 45.8% to 48.8% higher than for postdocs in academia. Median starting salary for PSE postdocs, adjusted for inflation to 2018 values [34], varies across sectors: $46,610 (non-U.S. positions), $48,000 (academic), $53,000 (non-profit), $70,000 (government), and $71,400 (industry). LS postdocs have less of a salary differential, with 19.0% to 35.4% higher median starting salaries in government and industry than in academia. Median starting salary for LS postdocs (2018 values) also varies across sectors: $44,554 (academic), $45,600 (non-U.S. positions), $46,800 (non-profit), $53,000 (government), and $60,320 (industry). We ran a linear regression on postdoc starting salary (see S2 Table in S2 Appendix), finding that sex, race, discipline, Carnegie classification, and sector at graduation were statistically significant in predicting initial postdoc salary at graduation. However, the variance explained by the model is less than 10%.

**Fig 3:**
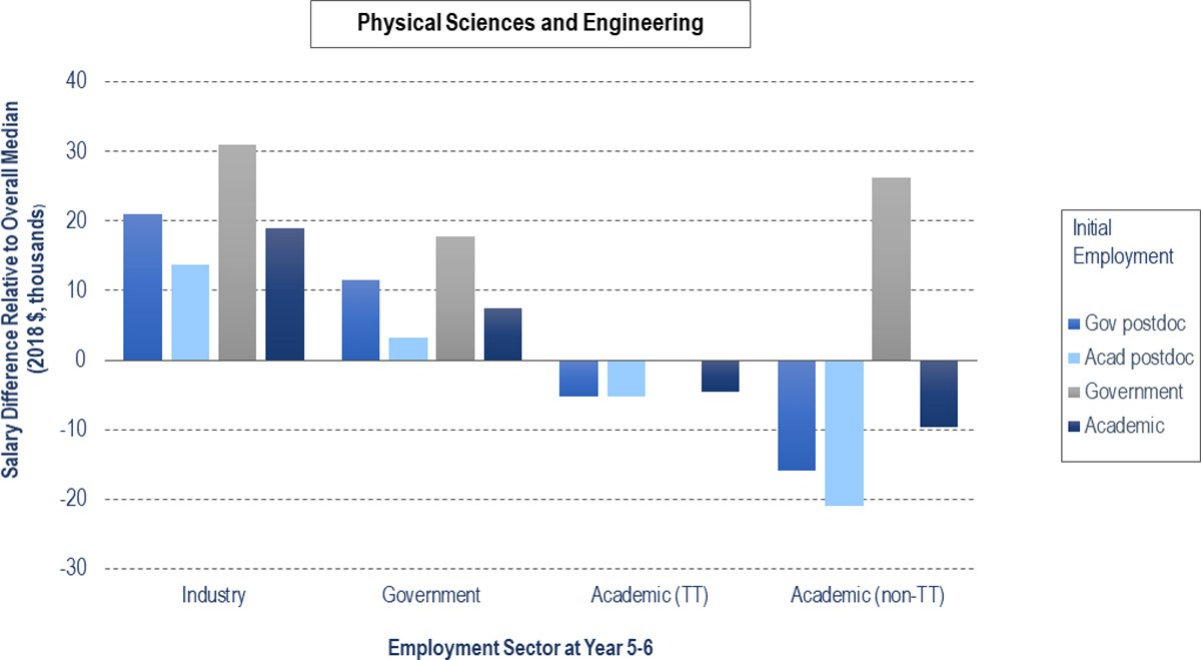
Median salary comparison at Year 5–6 for physical sciences and engineering by initial employment type and sector.

**Fig 4:**
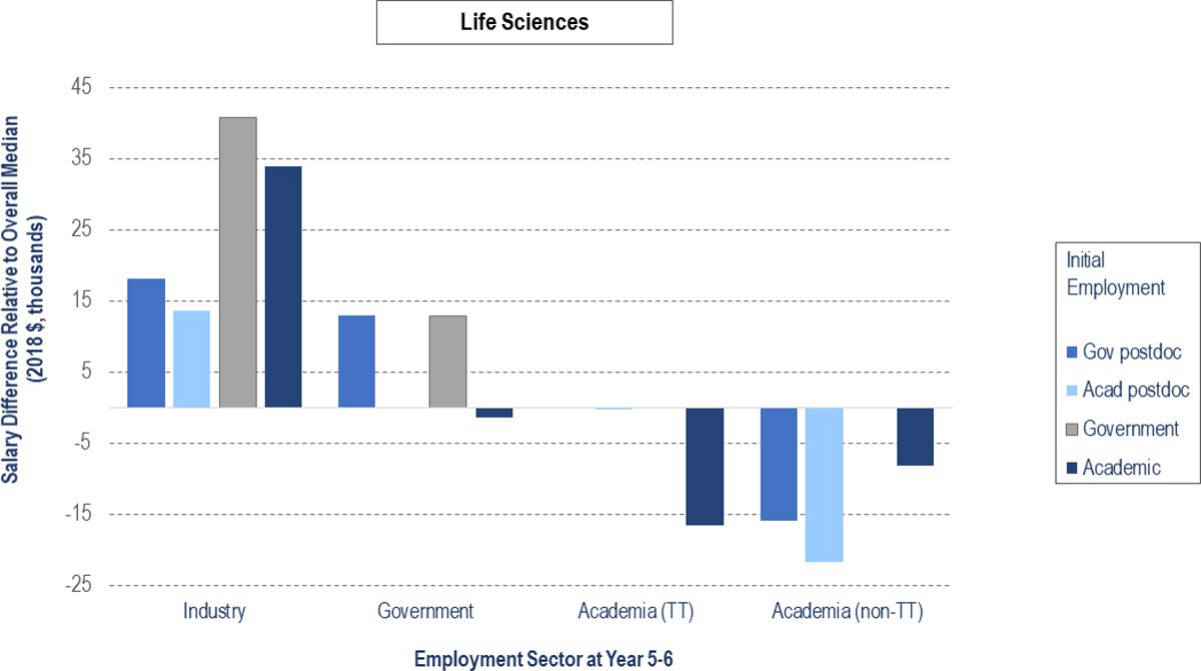
Median salary comparison at Year 5–6 for life sciences by initial employment type and sector.

Figs 3 and 4 compares median salaries for permanent employment at 5–6 years after earning their doctorate in the largest sectors for PSE (Fig 3) and LS (Fig 4) PhDs who start in government and academic positions. The y-axis is scaled according to the overall median salary ($94,500 for PSE, $81,600 for LS), displaying relative salary differences for each sector. In many categories, the final salaries of government postdocs are comparable to those who started in government and academic permanent employment at graduation. PSE government postdocs fare better than PSE academic postdocs who end up in permanent industry (by $7,350), government (by $8,430), and non-tenure track academic positions (by $5,145). For tenure-track positions, former PSE government and academic postdocs have equivalent median salaries ($89,250). LS government postdocs fare better than LS academic postdocs who end up in permanent industry (by $4,545), government (by $12,900), tenure-track faculty positions (by $255), and non-tenure-track academic positions (by $5,850). We ran a linear regression on permanent employment salary at Year 5–6 (see S3 Table in S2 Appendix), finding that race, discipline, and sector at Year 5–6 were statistically significant in predicting salary at Year 5–6. However, the variance explained by the model is less than 1%.

Display of salary gaps and surpluses when compared to overall median salary (zero on y-axis, $94,500, PSE) for individuals who held initial employment in government or academic sectors at graduation and employment at Year 5–6 in industry, government, or academic sectors (n = 1392, PSE). Bars corresponding to values of n < 5 are not included.

Display of salary gaps and surpluses when compared to overall median salary (zero on y-axis, $81,600, LS) for individuals who held initial employment in government or academic sectors at graduation and employment at Year 5–6 in industry, government, or academic sectors (n = 1244). Bars corresponding to values of n < 5 are not included. In the life sciences, the median salaries for individuals who held initial government postdocs and academic tenure-track positions at Year 5–6 and individuals who held initial academic postdocs and government positions at Year 5–6 are $81,600, which is the same as the overall median salary (zero on y-axis) and do not appear when plotted.

## Discussion

One of our main analytical goals was to develop a more comprehensive overview of postdoctoral career pathways, including trends in non-academic sectors and across science and engineering fields. We provide empirical data that supports conventional wisdom about the duration and salary of government and industry postdocs. Within each employment sector, we found that PSE postdocs are shorter in duration and receive higher starting salaries than LS postdocs. These field differences become more pronounced overall with the higher percentages of PSE postdocs employed in government and industry. Within the past decade, there were almost equal numbers of new LS (n = 40,975) and PSE (n = 40,565) postdocs among recent graduates, emphasizing the prevalence of postdoc positions outside of the biological sciences. Although LS doctorates enter postdoc roles at higher rates as compared to PSE doctorates, almost half of PSE graduates took a postdoc as their initial employment. Our findings counter common narratives about career opportunities and potential motivations for postdoctoral employment and introduce important implications for advising PhD researchers about different employment sectors.

Across each employment sector, LS postdocs are longer in duration than those in PSE. Within each field, industry and government have the shortest length postdocs compared to other sectors, particularly the academic sector. Academic postdocs in LS are almost 8 months (government) and 20 months (industry) longer, whereas academic postdocs in PSE are almost 3 months (government) and 14 months (industry) longer. With higher percentages of PSE postdocs in government and industry sectors, the disciplinary differences in postdoc length become more pronounced. Shorter or longer postdocs can be beneficial for doctorates, dependent on their field, career goals, and reasons for taking postdoctoral employment. Longer postdocs allow doctorates to engage in research projects, develop research skills, and write publications over a longer time period, which can be helpful for future job preparation. However, postdocs are often paid less than permanent employees, with longer postdoc duration contributing to a greater salary gap. Postdoc duration may be dependent on the grants and funding available in the different sectors, resulting in shorter industry postdocs if industry is less dependent on multi-year grants.

Overall, many reasons for choosing a postdoc position were similar between fields and sectors. Primarily, respondents chose postdoctoral employment for further training or viewed taking a postdoc as expected within their field, indicating that postdocs are part of the normal post-PhD pathway in the sciences and viewed as such by recent PhD recipients. The least frequent reason for employment in a postdoc position was that there was no other employment available, which refutes the common narrative that individuals only take postdocs when they have no other job offers. However, our finding that 20% of continuing postdocs indicated no other employment was available is not negligible, particularly since women and URG STEM PhDs are most likely to have no job prospects upon graduation [35], and non-U.S.-born Asian doctorates are more likely to take postdoc positions because there are no options available [36]. Additionally, this number may be under-reported if respondents did not feel comfortable disclosing that lack of availability of positions influenced their current employment. With the continued growth of postdoctoral positions [2], and the expectations of recent doctorates to take such roles, the training period involved with a science PhD extends closer to a decade. This requisite continued training may further decrease the accessibility of science careers for a variety of groups, such as those of lower socioeconomic status (SES), women, and URG doctorates.

We found that 23.0% of LS and 33.2% of PSE postdocs were employed in tenure-track faculty positions within 5–6 years following degree completion. Although our results suggest that PSE postdocs secure tenure-track employment at higher rates than LS postdocs, we caution potential over-interpretations of this finding. We excluded respondents who remained in a postdoc at 5–6 years in our analysis, and LS postdocs tend to remain in postdocs longer, which might account for the lower percentage of LS postdocs transitioning to tenure-track positions at that time. Prior literature reports lower percentages of postdocs obtaining tenure-track faculty positions, namely 17 [8] to 21% [1]. Both studies analyzed over a 10 year period (rather than 5–6 years) and either focused on all science fields (including health and social sciences) [8] or solely biomedical PhDs [1], indicating that those staying in postdocs longer may be less likely to be in tenure-track roles and pointing to differences in employment trends between fields. As expected, transitioning from a postdoc to permanent employment occurred more frequently within the same sector than transitioning between sectors. However, a substantial number of academic postdocs transition to government or industry within 5–6 years in both fields, with LS academic postdocs less likely to obtain tenure-track positions and more likely to have other academic permanent positions as compared to PSE academic postdocs. We do not have data on respondents’ intended career path. However, to the extent that academic postdocs are viewed as the pathway to tenure-track roles, frequent movement to non-tenure-track positions raises the question of the extent to which academic postdocs should be thought of as a likely pathway to a tenure-track role.

The broad dispersal of government postdocs suggests this sector may be a good option for doctorates who are unsure about their permanent career goals, as it leaves open a range of future career options, including future tenure-track employment. The frequent movement between sectors within the first few years of employment suggests that careers should be discussed as dynamic and changing with science and engineering PhD students on the job market, rather than framed as overly dependent on their first job as a new PhD. Given the higher percentages of individuals who identified as female and URG doctorates in government postdocs, the inclusion of quality mentorship and unique research opportunities in those roles could be instrumental in encouraging individuals identifying as female and URG doctorates who are U.S. citizens or permanent residents to stay in science and engineering careers, including as future faculty. Movement between sectors could be occurring for a variety of reasons, including individuals’ changing career goals and job market considerations (i.e., what positions are available). Doctorates with citizenship outside of the U.S. often have additional restrictions on what employment they can hold.

Both LS and PSE non-academic postdoc median salaries are higher than those in academia, i.e., in industry and government. Academic postdoc salaries are comparable between the two fields, with slightly higher salaries for those in PSE (by $3,446). However, the postdoc salary gap between fields widens for non-academic postdocs, with greater salaries for PSE in industry (by $11,080) and government (by $17,000). Within each field, for postdocs who transitioned to permanent positions within 5–6 years, government postdocs had higher or equal permanent employment salaries compared to academic postdocs across all end sectors. In their permanent employment at 5–6 years, government postdocs also had higher salaries than those entering academic permanent employment immediately following the PhD across several end sectors, such as industry (PSE), government (PSE and LS), and academic tenure-track (LS). Thus, government postdocs do not necessarily follow the persistent salary gap trends between postdocs and non-postdocs identified in prior studies [1, 21]. However, across each end sector, median salaries of LS government postdocs were less than those of PSE academic postdocs. Before entering the job market, science PhDs should consider the long-term salary implications of postdoc sector for both their intended and alternative career plans. The postdoc disciplinary salary gap becomes a serious sex issue, as individuals who identified as female are in life science postdocs at much higher rates than physical sciences or engineering postdocs (Table 3).

These results reinforce the need to reconsider how we advise PhDs about career paths, as has been argued previously [2, 27, 37]. Graduate students need more information about nonacademic careers, as advisors tend to be ignorant of or biased against career paths other than tenure track faculty at research-intensive institutions [2]. Much of the published research and advice about postdoc career paths focuses on life sciences, but there are different patterns for the physical sciences and engineering as we found in our analysis. U.S. government postdocs should be further considered as beneficial early-career options for eligible PSE PhD students because of their higher salaries and opportunity to move between a variety of sectors, including future tenure-track faculty roles. The benefits of government postdocs in LS are less clear, with similar salary implications but less broad dispersion between sectors. Given the value of U.S. government postdoctoral positions to the training of PhD scientists and engineers in all areas of the economy, national laboratories might consider initiatives to expand the number of postdoc positions, alone or in collaboration with other organizations. There are a few existing initiatives that encourage the consideration of non-academic pathways and help prepare for such roles for both doctoral students (e.g., [38]) and postdocs (e.g., [6]). Future work should involve expanding such programs, as well as examining how the postdoctoral workforce and trajectory has been impacted in recent years by the ongoing pandemic and the resulting new labor market conditions.

## Supporting information

S1 Appendix Logistic regression on sector movement. (DOCX)

S2 Appendix Linear regressions of salary at graduation and at Year 5–6. (DOCX)

## References

[1] Kahn S, Ginther DK. The impact of postdoctoral training on early careers in biomedicine. Nature biotechnology. 2017;35(1):90. doi: 10.1038/nbt.3766 28072769

[2] Institute of Medicine. The Postdoctoral Experience Revisited. Washington, DC: The National Academies Press; 2014. 25590106

[3] Cantwell B, Taylor BJ. Rise of the science and engineering postdoctorate and the restructuring of cademic research. The Journal of Higher Education. 2015;86(5):667–96.

[4] National Center for Science and Engineering Statistics. Survey of Graduate Students and Postdoctorates in Science and Engineering. 2018.

[5] Åkerlind G. Postdoctoral research positions as preparation for an academic career. International Journal for Researcher Development. 2009;1(1):84–96.

[6] Hayter CS, Parker MA. Factors that influence the transition of university postdocs to non academic scientific careers: An exploratory study. Research Policy. 2019;48(3):556–70.

[7] Sauermann H, Roach M. Why pursue the postdoc path? Science. 2016;352(6286):663–4. doi: 10.1126/science.aaf2061 27151854

[8] Andalib MA, Ghaffarzadegan N, Larson RC. The postdoc queue: A labour force in waiting. Systems research and behavioral science. 2018;35(6):675–86.

[9] Schillebeeckx M, Maricque B, Lewis C. The missing piece to changing the university culture. Nature biotechnology. 2013;31(10):938–41. doi: 10.1038/nbt.2706 24104758

[10] Stephan PE. How economics shapes science: Harvard University Press Cambridge, MA; 2012.

[11] Pavlidis I, Petersen AM, Semendeferi I. Together we stand. Nature Physics. 2014;10:7002.

[12] Cantwell B, Lee J. Unseen workers in the academic factory: Perceptions of neoracism among international postdocs in the United States and the United Kingdom. Harvard Educational Review. 2010;80(4):490–517.

[13] Gibbs KD Jr, McGready J, Griffin K. Career development among American biomedical postdocs. CBE—Life Sciences Education. 2015;14(4):ar44. doi: 10.1187/cbe.15-03-0075 26582238 PMC4710405

[14] Shauman KA. Gender differences in the early employment outcomes of STEM doctorates. Social Sciences. 2017;6(1):24.

[15] McConnell SC, Westerman EL, Pierre JF, Heckler EJ, Schwartz NB. Career choice, gender, and mentor impact: results of the US national postdoc survey. bioRxiv. 2018:355511. 10.7554/eLife.40189 PMC6298783 30561332

[16] Chen S, McAlpine L, Amundsen C. Postdoctoral positions as preparation for desired careers: a narrative approach to understanding postdoctoral experience. Higher Education Research & Development. 2015;34(6):1083–96.

[17] Miller JM, Feldman MP. Isolated in the lab: Examining dissatisfaction with postdoctoral appointments. The Journal of Higher Education. 2015;86(5):697–724.

[18] Su X. Postdoctoral training, departmental prestige and scientists’ research productivity. The Journal of Technology Transfer. 2011;36(3):275–91.

[19] Holden C. General contentment masks gender gap in first AAAS salary and job survey. American Association for the Advancement of Science; 2001. doi: 10.1126/science.294.5541.396 11598304

[20] Athanasiadou R, Bankston A, Carlisle M, Niziolek CA, McDowell GS. Assessing the landscape of US postdoctoral salaries. Studies in Graduate and Postdoctoral Education. 2018.

[21] Main JB, Wang Y. Is postdoctoral training linked to faculty careers and higher salaries in engineering fields? American Society for Engineering Education Annual Conference; Tampa, FL2019.

[22] Main JB, Wang Y, Tan L. The career outlook of engineering PhDs: Influence of postdoctoral research positions on early career salaries and the attainment of tenure-track faculty positions. Journal of Engineering Education. 2021;110(4):977–1002.

[23] Smith-Doerr L. Stuck in the middle: Doctoral education ranking and career outcomes for life scientists. Bulletin of science, technology & society. 2006;26(3):243–55.

[24] Yang L, Webber KL. A decade beyond the doctorate: The influence of a US postdoctoral appointment on faculty career, productivity, and salary. Higher Education. 2015;70(4):667–87.

[25] Gibbs KD Jr, Griffin KA. What do I want to be with my PhD? The roles of personal values and structural dynamics in shaping the career interests of recent biomedical science PhD graduates. CBE–Life Sciences Education. 2014;12(4):711–723. 10.1187/cbe.13-02-0021 PMC3846521 24297297

[26] Canetto SS, Trott CD, Winterrowd EM, Haruyama D, Johnson A. Challenges to the choice discourse: Women’s view of their family and academic-science career options and constraints. Journal of Feminist Family Therapy. 2017;29(1–2):4–27.

[27] Dietz JS, Bozeman B. Academic careers, patents, and productivity: industry experience as scientific and technical human capital. Research policy. 2005;34(3):349–67.

[28] Woolston C. Industry: Open for business. Nature. 2016;537(7620):437–9.

[29] Kaplan K. Postdoc or not? Nature. 2012;483(7390):499–500. doi: 10.1038/nj7390-499a 22442840

[30] Institute of Medicine. Enhancing the Postdoctoral Experience for Scientists and Engineers: A Guide for Postdoctoral Scholars, Advisers, Institutions, Funding Organizations, and Disciplinary Societies. Washington, DC: The National Academies Press; 2000. 31556982

[31] Polka JK, Krukenberg KA, McDowell GS. A call for transparency in tracking student and postdoc career outcomes. Molecular Biology of the Cell. 2015;26(8):1413–5. doi: 10.1091/mbc.E14-10-1432 25870234 PMC4395122

[32] National Science Foundation. Survey of earned doctorates. Alexandria, VA: National Science Foundation; 2020.

[33] National Science Foundation. Survey of doctorate recipients. Alexandria, VA: National Science Foundation; 2021. Report No.:https://www.nsf.gov/statistics/srvydoctoratework/.

[34] Commonfund Institute. Commonfund higher education price index: 2018 update. Wilton, CT: Commonfund Institute; 2018.

[35] Kinoshita T, Knight D, Borrego M, Bortz WW. Illuminating systematic differences in no job offers for STEM doctoral recipients. PLoS One. 2020;15(4):e0231567. doi: 10.1371/journal.pone.0231567 32348344 PMC7190089

[36] Huang Y, Cantwell B, Taylor B. Reasons for becoming a postdoc: Differences by race and foreign-born status. Teachers College Record. 2016;118(11):1–29.

[37] National Academies of Sciences Engineering and Medicine. Graduate STEM Education for the 21st Century. Consensus Study Report. Washington, DC: The National Academies Press; 2018. Report No.: 0309472733.

[38] Denton M, Borrego M, Chang CN, Boklage A, Arroyave R. Non-academic career pathways for engineering doctoral students: An evaluation of an NSF traineeship program. American Society of Engineering Education Annual Conference; Virtual 2020.

