# Supplementary material for: U.S. postdoctoral careers in life sciences, physical sciences and engineering: Government, industry, and academia
Source: PLoS One. 2022 Feb 2;17(2):e0263185. doi: 10.1371/journal.pone.0263185 (PMC8809557; doi:10.1371/journal.pone.0263185)
Supplement: S1 Appendix — (DOCX) [file pone.0263185.s001.docx]

**Appendix S1. Logistic regression on sector movement**

**Table S1. Logistic regression on sector movement**

|  | Coefficient (Standard Error) | Odds Ratio |
| --- | --- | --- |
| Intercept |  |  |
| GENDER (ref: Male) | -0.494 (0.092) | 0.610 |
| Female | -0.184 (0.098) | 0.832 |
| RACE (ref: White) |  |  |
| Asian | 0.371 (0.109) *** | 1.449 |
| URG | -0.027 (0.141) | 0.973 |
| DISCIPLINE (ref: Physical sciences and engineering) |  |  |
| Life sciences | 0.158 (0.099) | 1.171 |
| CARNEGIE CLASSIFICATION (ref: R1) |  |  |
| Non-R1 | 0.047 (0.134) | 1.048 |
| START SECTOR (ref: Academic) |  |  |
| Government | 1.128 (0.142) *** | 3.091 |
| Industry | -0.973 (0.347) ** | 0.378 |
| McFadden R^2^ | 0.0654 |  |
| n | 1922 |  |

*p < 0.05, **p < 0.01, ***p < 0.001

All variables were categorized as previously described in the Methods in the main text. We included one new independent variable, *Carnegie classification*. The SED code PHDCARN indicated the Carnegie classification of respondents’ doctoral institution. We categorized participants as receiving a doctoral degree from an R1 institution if they selected *research universities (very high research activity)* and as receiving a doctoral degree from a non-R1 institution if they selected any of the other options. We also included a new dependent variable, *switch sector*. We coded this variable as 1 if the sector at graduation and sector at Year 5-6 were not the same (i.e., Academic at graduation and Industry at Year 5-6). *Switch sector* was coded as 0 if the sector at graduation and sector at Year 5-6 were the same (i.e., Government at graduation and Government at Year 5-6).

We used the same participant dataset as in the *movement between sectors* analysis; namely, respondents in LS and PSE who took a postdoc at graduation and held permanent employment at Year 5-6 in the sectors of Academic, Government, and Industry at both timepoints. All variables in the logistic regression were dummy coded, with the reference category for each group included in Table S1. Within the model, three variables are statistically significant: Asian, Start Government, and Start Industry. Participants who identified as Asian were 45% more likely to switch sectors (odds ratio 1.449) than participants who identified as White. Participants who started in government were 209% more likely to switch (odds ratio 3.091) than those who started in academic, while participants who started in industry were 62% less likely to switch (odds ratio 0.378) than those who started in academic. The total variance explained by the logistic regression model is 6.54%.
