# Supplementary material for: U.S. postdoctoral careers in life sciences, physical sciences and engineering: Government, industry, and academia
Source: PLoS One. 2022 Feb 2;17(2):e0263185. doi: 10.1371/journal.pone.0263185 (PMC8809557; doi:10.1371/journal.pone.0263185)
Supplement: S2 Appendix — (DOCX) [file pone.0263185.s002.docx]

**Appendix S2: Linear regressions of salary at graduation and at Year 5-6**

**Table S2. Linear regression of postdoc salary at graduation**

|  | Outcome: Salary (2018 dollars) |
| --- | --- |
|  | Coefficient (Standard Error) |
| Intercept | 51206.8 (173.9) *** |
| GENDER (ref: Male) |  |
| Female | -510.1 (214.8) * |
| RACE (ref: White) |  |
| Asian | 939.2 (433.0) * |
| URG | 100.5 (347.5) |
| DISCIPLINE (ref: Physical sciences and engineering) |  |
| Life sciences | -4766.9 (213.7) *** |
| CARNEGIE CLASSIFICATION (ref: R1) |  |
| Non-R1 | -1818.3 (271.1) *** |
| START SECTOR (ref: Academic) |  |
| Government | 16732.8 (335.3) *** |
| Industry | 22525.2 (636.0) *** |
| Non-profit | 4416.4 (539.5) *** |
| Non-U.S. | -22.9 (394.6) |
| Adjusted R^2^ | 0.0736 |
| n | 56,633 |

*p < 0.05, **p < 0.01, ***p < 0.001

All variables were categorized as previously described in the Methods in the main text. We included one new independent variable, *Carnegie classification*. The SED code PHDCARN indicated the Carnegie classification of respondents’ doctoral institution. We categorized participants as receiving a doctoral degree from an R1 institution if they selected *research universities (very high research activity)* and as receiving a doctoral degree from a non-R1 institution if they selected any of the other options. The dependent variable in the model is postdoc *salary at graduation*, adjusted for inflation to 2018 dollars.

We used the same participant dataset as in the *initial salaries* analysis; namely, respondents in LS and PSE who took a postdoc at graduation in the sectors of Academic, Government, Industry, Non-profit, and Non-U.S. and filled out their starting salary information. All independent variables in the linear regression were dummy coded, with the reference category for each group included in Table S2. Within the model, seven variables are statistically significant: Female, Asian, Life Sciences, Non-R1, Start Government, Start Industry, and Start Non-profit. Participants who identify as female are likely to be paid less as compared to participants who identify as male, while participants who identify as Asian are likely to be paid more as compared to participants identifying as White. Participants in the life sciences are likely to be paid less compared to those in the physical sciences and engineering, and participants receiving PhDs from non-R1 institutions are likely to be paid less than those from R1 institutions. Participants starting postdocs in government, industry, and non-profit sectors are likely to be paid more than participants starting postdocs in the academic sector. The total variance explained by the logistic regression model is 7.36%.

**Table S3. Linear regression of permanent employment salary at Year 5-6**

|  | Outcome: Salary (2018 dollars) |
| --- | --- |
|  | Coefficient (Standard Error) |
| Intercept | 148391.8 (10302.6) *** |
| GENDER (ref: Male) |  |
| Female | **-**20557.1 (9084.9) * |
| RACE (ref: White) |  |
| Asian | -9334.4 (10176.2) |
| URG | 10790.7 (12727.3) |
| DISCIPLINE (ref: Physical sciences and engineering) |  |
| Life sciences | -21428.8 (9467.0) * |
| CARNEGIE CLASSIFICATION (ref: R1) |  |
| Non-R1 | -11501.8 (11501.0) |
| START SECTOR (ref: Industry) |  |
| Academic | 588.6 (15327.9) |
| Government | -2729.2 (18380.3) |
| EMPLOYMENT TYPE AT GRADUATION  (ref: Permanent) |  |
| Postdoc | -415.1 (11390.1) |
| END SECTOR (ref: Industry) |  |
| Academic | -37574.9 (11094.1) *** |
| Government | -21831.0 (16979.9) |
| Adjusted R^2^ | 0.0077 |
| n | 3,481 |

*p < 0.05, **p < 0.01, ***p < 0.001

All variables were categorized as previously described in the Methods in the main text. The categorization of the new variable *Carnegie classification* is described above. The dependent variable in the model is s*alary at Year 5-6*, adjusted for inflation to 2018 dollars.

We used a similar participant dataset as in the *movement between sectors* analysis; namely, respondents in LS and PSE who took a postdoc or permanent employment at graduation and held permanent employment at Year 5-6 in the sectors of Academic, Government, and Industry at both timepoints. Unlike the *movement between sectors* analysis, which includes only postdocs at graduation, we also include respondents who took permanent employment at graduation in the linear regression. All independent variables in the linear regression were dummy coded, with the reference category for each group included in Table S3. Within the model, three variables are statistically significant: Female, Life Sciences, and End Academic. Participants who identify as female are likely to be paid less as compared to participants who identify as male, and participants in the life sciences are likely to be paid less as compared to those in physical sciences and engineering. Participants in academic permanent employment at Year 5-6 are likely to be paid less than participants in industry permanent employment at Year 5-6. The total variance explained by the logistic regression model is 0.77%.
